# Human cathelicidin, LL-37, inhibits respiratory syncytial virus infection in polarized airway epithelial cells

**DOI:** 10.1186/s13104-015-1836-y

**Published:** 2016-01-05

**Authors:** Jennifer L. Harcourt, Melissa McDonald, Pavel Svoboda, Jan Pohl, Kathleen Tatti, Lia M. Haynes

**Affiliations:** National Center for Immunization and Respiratory Diseases, Division of Viral Diseases, Gastroenteritis and Respiratory Viruses Laboratory Branch, Centers for Disease Control and Prevention (CDC), 1600 Clifton Road NE, Mailstop A-34, Atlanta, GA 30333 USA; Biotechnology Core Facility Branch, Division of Scientific Resources, Centers for Disease Control and Prevention, Atlanta, GA USA

**Keywords:** LL-37, Cathelicidin, Respiratory syncytial virus, Air–liquid interface, Calu-3

## Abstract

**Background:**

Respiratory syncytial virus (RSV) is a major cause of severe lower respiratory tract illness in young children worldwide. Treatment options for severe RSV disease remain limited and the development of therapeutic treatment strategies remains a priority. LL-37, a small cationic host defense peptide involved in anti-inflammatory and anti-bacterial responses, reduces replication of or infection by multiple viruses, including influenza virus, in vitro, and protects against lethal challenge with influenza virus in vivo. LL-37 also protects against RSV infection of HEp-2 cells in vitro; however, HEp-2 are not reflective of polarized airway epithelial cells and respond differently to RSV infection. An air–liquid interface (ALI) Calu-3 model that more closely mimics the human airway epithelium was established. Using this in vitro model, the effectiveness of LL-37 in preventing RSV infection and replication was examined.

**Results:**

LL-37, when pre-incubated with virus prior to RSV infection (prophylactic), significantly reduced the level of viral genome detected in infected Calu-3 cells, and decreased chemokine expression associated with RSV infection in vitro. In contrast, therapeutic treatment of RSV-infected ALI Calu-3 at 24 h and 3 days post-infection had minimal impact on RSV infection.

**Conclusions:**

Differences in the efficacy of LL-37 at reducing RSV infection under prophylactic and therapeutic conditions may in part be ascribed to differences in the method of peptide exposure. However, the efficacy of LL-37 at reducing RSV infection under prophylactic conditions indicates that further studies examining the efficacy of LL-37 as a small peptide inhibitor of RSV are warranted.

## Findings

Respiratory syncytial virus (RSV) is a major cause of lower respiratory tract illness in infants and children and of serious disease in elderly and immune compromised patients [1–3]. RSV infection results in substantial morbidity and hospitalizations each year [4, 5], and treatment for RSV associated illness is limited. There is currently no safe and effective licensed vaccine against RSV, and immunoprophylaxis with palivizumab is indicated to reduce the incidence of RSV-associated disease in high-risk infants [6]. Treatment of acute RSV disease remains primarily supportive in nature [7]. Aerosolized ribivarin, a synthetic nucleotide analog, may be considered for use in severe RSV disease in hospitalized patients or in those who are at risk for severe disease; however, due to its expense, delivery method, and toxicity, its use is limited [8]. Several small molecule inhibitors have been evaluated for treatment of RSV [9–11], but none have yet been reported to be effective in humans. Thus, the development of alternative therapeutic strategies remains important.

The cationic peptide cathelicidin, LL-37, is an important part of the early innate immune response to bacterial infection. LL-37 is the predominant active cleavage product of the cationic host defense peptide hCAP18, and its expression, upregulated in response to inflammation and bacterial and viral infection, is detectable in multiple cell types, including neutrophils, epithelial cells, and macrophages [12]. Initially characterized as an anti-microbial peptide, LL-37 also demonstrates anti-viral activity. In vitro, LL-37 inhibits replication of human immunodeficiency virus -1 (HIV-1) in peripheral blood mononuclear cells [13], reduces vaccinia virus plaque formation and mRNA expression [14], and reduces infectious virus following herpes simplex virus -1 (HSV-1) and adenovirus-19 infection in A549 cells [15]. LL-37 has potent in vitro and in vivo anti-viral activity against influenza virus. LL-37 therapeutic treatment reduced mortality, virus titers, and the levels of cytokine expression in the lungs of mice challenged with a lethal strain of influenza virus [16]. LL-37 is also effective at reducing the number of RSV-infected HEp-2 cells, and at reducing the spread of RSV infection in HEp-2 in vitro [17].

HEp-2 cells, derived from a human laryngeal carcinoma, are often used to propagate RSV, and to study human epithelial cellular responses to RSV infection. However, HEp-2 cells do not polarize or differentiate, and in contrast to a polarized, differentiated model of normal human bronchial epithelial cells [18, 19], HEp-2 form large cytopathic effect (CPE) following RSV infection, indicating that the HEp-2 cell line is not an ideal model system for characterizing in vivo human cellular response to RSV infection. Previous studies have demonstrated that liquid covered cultures of polarized Calu-3 cells (LCC Calu-3) are susceptible to RSV infection [20, 21]. However, these cells are cultured with medium at both the apical and basolateral surfaces. To more closely mimic the physiology of the human airway epithelium, air–liquid interface cultures of Calu-3 (ALI Calu-3), shown to exhibit morphological characteristics similar to differentiated, polarized normal human bronchial epithelial cells (NHBE) [18], were derived from liquid-covered Calu-3 (LCC Calu-3) cultures. Before examining the ability of LL-37 to inhibit RSV infection of Calu-3 cells, the susceptibility of ALI Calu-3 to RSV strain A2 (RSV-A2) infection was compared to that of LCC Calu-3, examining relative viral genome levels by qRT-PCR, reported as genome equivalents/ml, and the production of infectious virus by plaque assay, reported as PFU/ml. Similar to LCC Calu-3, ALI Calu-3 were susceptible to RSV-A2 infection at the apical surface, with viral genome or infectious virus detected as early as 3 days post-infection (pi), and little infectious virus detected in the basolateral compartment of cultures following infection (data not shown). Identical relative levels of viral genome in RSV-A2 infected ALI Calu-3 and LCC Calu-3 were observed at 3 days (6.2 × 10^4^ genome equivalents/ml RSV-A2 infected ALI-Calu-3 and 8.6 × 10^4^ genome equivalents/ml RSV-A2 infected LCC Calu-3; p = 0.073) and 1 week pi (1.0 × 10^5^ genome equivalents/ml RSV-A2 infected ALI-Calu-3 and 1.7 × 10^5^ genome equivalents/ml RSV-A2 infected LCC Calu-3; p = 0.20) indicating that there are no differences in susceptibility to infection between ALI and LCC Calu-3 models. Though viral genome was detectable as early as 3 days pi, the production of infectious virus by RSV-A2 infected ALI Calu-3 was not consistently detectable from all replicates until 7 days pi, and the level of viral genome production reached a plateau at day 7 pi. Thus, studies were performed at 7 days pi. Taken together, ALI Calu-3 maintained a stable ALI and can be used as an in vitro model for RSV infection of human airway epithelium. Thus, the effectiveness of LL-37 as a potential prophylactic and therapeutic treatment against RSV infection were examined in ALI Calu-3.

**Fig. 1:**
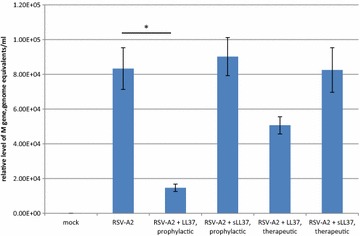
LL-37 reduces RSV-A2 infection of and replication in ALI Calu-3. ALI—cultured Calu-3 cells were infected at the apical surface with RSV-A2 at MOI = 1, or treated prophylactically or therapeutically with 50 μg/ml LL-37 or sLL-37. Prophylactic treatment was defined as a 1 h co-incubation of virus with peptide immediately prior to infection. Therapeutic treatment was defined as inclusion of peptide in the basolateral medium, beginning 24 h pi, with replacement of medium and peptide at 3 days pi. Total cellular RNA was isolated from five replicates per infection condition at 7 days pi, and the relative level of RSV-M gene was determined by qRT-PCR. Data is presented as the mean relative level of RSV-M gene, in genome equivalents/ml, of five replicates ± SD. *Asterisk* indicates p ≤ 0.05 as determined by unpaired two-tailed analysis. The data presented in this figure is representative of three independent experiments

Peptides LL-37 (LLGDFFRKSKEKIGKEGKRIVQRIKDFLRNLVPRTES) and an LL-37 analog having a “scrambled” sequence (RSLEGTDRFPFVRLKNSRKLEFKDIKGIKREQFVKIL; sLL37 control peptide) were synthesized as previously described [16]. To assess in vitro antiviral effects of these peptides, RSV-A2 was exposed to 50 μg/ml peptides for 1 h at 37 °C in serum-free Eagle’s minimum essential medium (EMEM) prior to apical infection of ALI Calu-3, (prophylactic treatment, performed using undiluted peptide-RSV A2 incubated reaction), or ALI Calu-3 were infected at the apical surface with RSV-A2, and peptides were added to the basolateral medium of ALI Calu-3 24 h after infection and replenished 3 days pi (therapeutic treatment). Dose response studies performed in monolayer cultured, non-polarized Calu-3 demonstrated that prophylactic administration of 50 μg/ml of LL-37 effectively inhibited the release of infectious virus from cells, whereas tenfold lower doses of LL-37 did not inhibit the release of infectious virus from infected cells (data not shown). In ALI Calu-3, pre-incubation of RSV-A2 with 50 μg/ml LL-37 under prophylactic conditions resulted in a range of 60–92 % reduction in the amount of viral genome detected in infected cells at 7 days pi (Fig. 1) as compared to untreated, infected cells (p = 0.00043) or sLL-37 treated, infected cells (p = 0.00022). Therapeutic treatment of RSV-A2—infected ALI Calu-3, in which 50 μg/ml LL-37 was added to the basolateral compartment of infected cells 24 h pi and replenished at 3 days pi, was associated with a 39 % reduction in the amount of viral genome detected at 7 days pi (p = 0.054). In contrast, prophylactic or therapeutic treatment with the control scrambled LL-37, was not associated with a change in the level of viral genome detected in infected ALI Calu-3, demonstrating the specificity of the LL-37 peptide sequence at inhibiting RSV replication. Consistent with previous studies, RSV-A2 infection of polarized Calu-3 did not impact the trans-epithelial electrical resistance (TEER) of infected Calu-3 cells at 7 days pi (Table 1, [21]). At the time point examined, treatment with LL-37 and sLL-37 did not impact the TEER of infected ALI Calu-3, indicating that the concentration of peptides used in this study was not detrimental to the polarized nature of the cultures (Table 1). Though the TEER at day 7 pi was below 1000 Ω × cm^2^, the ALI cultures retained their ALI.

**Table 1:** Trans-epithelial electrical resistance of ALI Calu-3 at 7 days post-infection

Treatment	Untreated^a^	Prophylactic	Therapeutic
Mock-infected	392 ± 16 (range 376–451)	–	‒
RSV-A2	445 ± 152 (range 421–1098)	‒	‒
RSV-A2 + LL-37	‒	520 ± 105 (range 315–886)	313 ± 84 (range 184–594)
RSV-A2 + sLL-37	‒	560 ± 126 (range 289–936)	316 ± 114 (range 221–864)

The trans-epithelial electrical resistance (TEER) of five wells per treatment condition was evaluated at day 7 post-infection, and is presented as median Ω × cm^2^ ± SEM of five individual wells per treatment condition, with the range of TEER measurements indicated in parentheses. No significant differences between prophylactic and therapeutic treatments or between mock-infected and infected treatment conditions were found using unpaired two-tailed statistical analysis. The data presented in this table is representative of three independent experiments

^a^ All experimental conditions were performed as one experiment; mock infected and RSV-A2 infected controls in the absence of peptide were performed simultaneously alongside prophylactic and therapeutic peptide treatments of infected ALI Calu-3

**Fig. 2:**
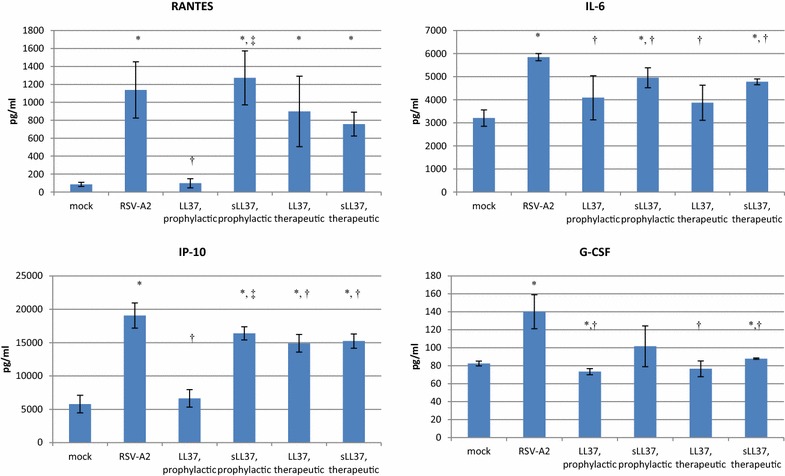
Prophylactic treatment with LL-37 reduces chemokine expression associated with RSV-A2 infection. ALI—cultured Calu-3 cells were infected at the apical surface with RSV-A2 at MOI = 1, or treated prophylactically or therapeutically with 50 μg/ml LL-37 or sLL-37. Prophylactic treatment was defined as a 1 h co-incubation of virus with peptide immediately prior to infection. Therapeutic treatment was defined as inclusion of peptide in the basolateral medium, beginning 24 h pi, with replacement of medium and peptide at 3 days pi. At 7 days pi, an apical wash was performed of all samples, and cytokine and chemokine expression was determined by 27-plex Bioplex assay of three samples per infection condition. Data is presented as mean pg/ml of each cytokine ± SD. *Asterisk*, p ≤ 0.05 compared to levels of expression from mock-infected ALI-Calu-3; *Dagger*, p ≤ 0.05 compared to levels of expression from RSV-A2—infected ALI-Calu-3; *Double dagger*, p ≤ 0.05 between LL-37 and sLL-37—treated, RSV-A2 infected ALI-Calu-3, as determined by unpaired two-tailed analysis. The data presented in this figure is representative of three independent experiments

RSV infection of airway epithelium is associated with induction of multiple cytokines and chemokines. To evaluate the impact of LL-37 treatment on cytokine and chemokine expression induced in response to RSV-A2 infection of ALI Calu-3, a 30 min wash of the apical surface of infected cells with EMEM + 10 % heat-inactivated fetal bovine serum was obtained 7 days pi, and cytokine and chemokine expression levels were determined using a Bioplex Cytokine Assay (BioRad) according to the manufacturer’s directions. RSV-A2 infection of ALI Calu-3 was consistently associated with a statistically significant (p ≤ 0.05) increase in the apical release of IP-10 and RANTES (Fig. 2, p ≤ 0.05 compared to mock-infected ALI Calu-3). The levels of IL-1ra, IL-4, IL-10, IL-13, IFNγ, MCP-1, PDGF-BB, bFGF and VEGF released from the apical surface of ALI Calu-3 did not differ following mock—or RSV-A2 infection at 7 days pi (data not shown). Finally, the levels of IL-1β, IL-2, IL-5, IL-7, IL-8, IL-9, IL-12p70, IL-15, IL-17, G-CSF, MIP-1α and -1β, TNFα, eotaxin, and GM-CSF released from the apical surface of ALI-Calu-3 did not demonstrate consistent changes between experiments following RSV-A2 infection (data not shown). In 2 of 3 replicate experiments, RSV-A2 infection was also associated with significant increase in the apical release of IL-6 (p = 0.0003) and G-CSF (p = 0.006)). LL-37 and sLL-37, used either under prophylactic or therapeutic conditions, reduced RSV-A2—induced expression of both of these cytokines. However, LL-37 reduced RSV-A2—induced IL-6 expression by 34–40 % (p = 0.035), whereas sLL37 reduced RSV-A2—induced IL-6 expression by 16–18 % (p = 0.029).

The induction of RANTES and IP-10 by RSV infection is consistent with previous studies in other cell lines [22, 23], and in plasma following RSV infection in children [24], however their function in modulating the immune response to RSV infection is unclear. The expression of RANTES and IP-10, the only chemokines whose expression was consistently increased at 7 days post infection with RSV-A2, were specifically reduced by prophylactic treatment of RSV-A2 with LL-37 (p = 0.005 and 0.001, respectively), and not by sLL37 (Fig. 2). In contrast, therapeutic treatment of RSV-A2—infected ALI Calu-3 with either LL-37 or sLL-37 was associated with a reduction in the levels of RSV-A2 associated levels of expression of IP-10 (p = 0.035 and 0.038, respectively), but neither peptide significantly impacted the induction of RANTES expression in response to RSV-A2 infection.

In our study, pre-incubation of RSV with LL-37 was more efficient than therapeutic treatment at reducing viral load and chemokine expression in RSV infected cells. These observations are consistent with previous studies, in which LL-37 reduced RSV-A2 infection of HEp-2 cells, when LL-37 was incubated with either cells or virus prior to infection, and also when administered simultaneously with RSV infection [17]. These observations suggest that LL-37 may be effective in part through direct interaction with RSV, and in part through interaction with and uptake by HEp-2 cells [17]. Similar to the results of our study, delayed treatment of HEp-2 cells ablated the ability of LL-37 to reduce RSV infection or spread in vitro [17]. Additionally, pre-incubation of influenza virus with LL-37 was more successful at neutralizing infection of MDCK and primary human tracheobronchial epithelial cells (HTBE) compared to treatment of infected cells with LL-37 after infection [25], in part due to direct interaction of LL-37 with influenza virus. Together, these studies suggest that LL-37 may have been more effective at reducing the amount of viral genome following RSV infection when used as a prophylactic agent instead of a therapeutic agent because pre-incubation of virus with LL-37 reduced the amount of infectious RSV available to infect ALI Calu-3. Differences in peptide uptake at the apical and basolateral surfaces may also be partly responsible for the observed differences in the ability of LL-37 to reduce viral replication when used under therapeutic treatment conditions. Currently, there is no evidence that endogenous hCAP-18/LL-37 is activated upon RSV infection, and endogenous expression of LL-37 by Calu-3 cells has not been demonstrated. Additional studies are required to determine whether treatment of Calu-3 with peptide, as opposed to pre-incubation with RSV-A2, inhibits RSV infection of ALI Calu-3, and whether the apical and basolateral surfaces of ALI Calu-3 differ in their ability to uptake LL-37 peptide.

Human cathelicidin LL-37 enhances TLR3—mediated signaling and IL-6, IL-10, and MCP-1 expression in the airway epithelial cell line BEAS-2B in response to rhinovirus infection [26]. The ability of TLR3 to mediate double-stranded RNA responses in BEAS-2B is due to endosomal co-localization of LL-37 and TLR3, and the presence of LL-37 in TLR3—containing endosomes is increased in the presence of dsRNA [27], an intermediate in the replication cycle of RSV. RSV infection of A549 and of human tracheal bronchial epithelial cells (hTBE) induces expression of TLR3 and upregulates NF-κB activation and cytokine expression in response to dsRNA in a PKR-dependent manner [28]. These observations contrast with our study, in which LL-37 was associated with reduced levels of cytokine and chemokine expression in response to infection. Previous studies have demonstrated that the amount of RSV strain A used to infect human epithelial kidney cells directly correlated with the level of CXCL8/IL-8 and RANTES produced in response to infection, and chemokine production in response to infection was dependent on TLR3 expression [29]. Thus, although TLR3 has been associated with increased cytokine and chemokine production in response to dsRNA and to RSV infection, the lower amount of viral RNA present in LL-37—treated ALI Calu-3 may at least in part be responsible for the lower levels of cytokine and chemokine production following RSV infection. However, the differences observed in cytokine and chemokine expression may also in part be due to the greater fusion activity and pathogenicity of RSV A compared to RSV-A2 [30–33].

The mechanism by which both LL-37 and sLL-37 reduce RANTES and IP-10 expression when used to therapeutically treat RSV—infected ALI Calu-3 in our studies is unclear. Recent studies have demonstrated apical, cytoplasmic, and basolateral expression of TLR3 in human airway epithelium, primary human airway epithelial cell cultures, and polarized BEAS-2B cells [34], suggesting that the ability of LL-37 to interact with TLR3 is not impacted by basolateral delivery of LL-37 as used in our studies. Cationic peptides, including poly-arginine, are able to activate TLR3 signaling in BEAS-2B cells [26], suggesting that at least some of the non-specific ability of sLL-37 to inhibit cytokine and chemokine expression in RSV-infected ALI Calu-3 may be due to the cationic nature of the peptide.

Our results show a reduction in intracellular viral genome and in the production of cytokines and chemokines associated with RSV strain A2 when LL-37 is used in a prophylactic regimen. These studies support further evaluation of LL-37 effectiveness against more pathogenic RSV strains and recent clinical isolates.

## Availability of supporting data

The data sets supporting the results of this article are included within this article.

## Abbreviations

RSV: respiratory syncytial virus
Pi: post-infection
LCC: liquid covered culture
ALI: air–liquid interface
TEER: trans-epithelial electrical resistance
MOI: multiplicity of infection
EMEM: Eagle’s modified essential medium

## References

[1] Hall CB, Walsh EE, Long CE, Schnabel KC (1991). Immunity to and frequency of reinfection with respiratory syncytial virus. J Infect Dis.

[2] Panitch HB (2001). Bronchiolitis in infants. Curr Opin Pediatr.

[3] Shay DK, Holman RC, Newman RD, Liu LL, Stout JW, Anderson LJ (1999). Bronchiolitis-associated hospitalizations among US children, 1980–1996. JAMA J Am Med Assoc.

[4] Stockman LJ, Curns AT, Anderson LJ, Fischer-Langley G (2012). Respiratory syncytial virus-associated hospitalizations among infants and young children in the United States, 1997–2006. Pediatr Infect Dis J.

[5] Zhou H, Thompson WW, Viboud CG, Ringholz CM, Cheng PY, Steiner C (2012). Hospitalizations associated with influenza and respiratory syncytial virus in the United States, 1993–2008. Clin Infect Dis.

[6] Tablan OC, Anderson LJ, Besser R, Bridges C, Hajjeh R, Cdc et al. Guidelines for preventing health-care–associated pneumonia, 2003: recommendations of CDC and the Healthcare Infection Control Practices Advisory Committee. MMWR Recommendations and reports: Morbidity and mortality weekly report recommendations and reports/Centers for Disease Control. 2004;53(RR-3):1–36. 15048056

[7] Verger JT, Verger EE (2012). Respiratory syncytial virus bronchiolitis in children. Crit Care Nurs Clin North Am.

[8] Policy Update: change in AAP guidance for use of synagis prophylaxis. In: Larry KP, editor. Red Book. American Academy of Pediatrics; 2012. p. 609–18.

[9] Cianci C, Meanwell N, Krystal M (2005). Antiviral activity and molecular mechanism of an orally active respiratory syncytial virus fusion inhibitor. J Antimicrob Chemother.

[10] Moore BP, Chung DH, Matharu DS, Golden JE, Maddox C, Rasmussen L (2012). (S)-N-(2,5-dimethylphenyl)-1-(quinoline-8-ylsulfonyl)pyrrolidine-2-carboxamide as a small molecule inhibitor probe for the study of respiratory syncytial virus infection. J Med Chem.

[11] Olszewska W, Ispas G, Schnoeller C, Sawant D, Van de Casteele T, Nauwelaers D (2011). Antiviral and lung protective activity of a novel respiratory syncytial virus fusion inhibitor in a mouse model. Eur Respir J.

[12] Durr UH, Sudheendra US, Ramamoorthy A (2006). LL-37, the only human member of the cathelicidin family of antimicrobial peptides. Biochim Biophys Acta.

[13] Bergman P, Walter-Jallow L, Broliden K, Agerberth B, Soderlund J (2007). The antimicrobial peptide LL-37 inhibits HIV-1 replication. Curr HIV Res.

[14] Howell MD, Jones JF, Kisich KO, Streib JE, Gallo RL, Leung DY (2004). Selective killing of vaccinia virus by LL-37: implications for eczema vaccinatum. J Immunol.

[15] Gordon YJ, Huang LC, Romanowski EG, Yates KA, Proske RJ, McDermott AM (2005). Human cathelicidin (LL-37), a multifunctional peptide, is expressed by ocular surface epithelia and has potent antibacterial and antiviral activity. Curr Eye Res.

[16] Barlow PG, Svoboda P, Mackellar A, Nash AA, York IA, Pohl J (2011). Antiviral activity and increased host defense against influenza infection elicited by the human cathelicidin LL-37. PLoS One.

[17] Currie SM, Findlay EG, McHugh BJ, Mackellar A, Man T, Macmillan D (2013). The human cathelicidin LL-37 has antiviral activity against respiratory syncytial virus. PLoS One.

[18] Grainger CI, Greenwell LL, Lockley DJ, Martin GP, Forbes B (2006). Culture of Calu-3 cells at the air interface provides a representative model of the airway epithelial barrier. Pharm Res.

[19] Zhang L, Peeples ME, Boucher RC, Collins PL, Pickles RJ (2002). Respiratory syncytial virus infection of human airway epithelial cells is polarized, specific to ciliated cells, and without obvious cytopathology. J Virol.

[20] Harcourt J, Haynes LM (2013). Establishing a liquid-covered culture of polarized human airway epithelial Calu-3 cells to study host cell response to respiratory pathogens in vitro. J Vis Exp.

[21] Harcourt JL, Caidi H, Anderson LJ, Haynes LM (2011). Evaluation of the Calu-3 cell line as a model of in vitro respiratory syncytial virus infection. J Virol Methods.

[22] Oshansky CM, Barber JP, Crabtree J, Tripp RA (2010). Respiratory syncytial virus F and G proteins induce interleukin 1alpha, CC, and CXC chemokine responses by normal human bronchoepithelial cells. J Infect Dis.

[23] Santini F (2015). Human respiratory syncytial virus and Th1 chemokines. La Clinica Terapeutica.

[24] Roe MF, Bloxham DM, Cowburn AS, O’Donnell DR (2011). Changes in helper lymphocyte chemokine receptor expression and elevation of IP-10 during acute respiratory syncytial virus infection in infants. Pediatr Allergy Immunol.

[25] Tripathi S, Tecle T, Verma A, Crouch E, White M, Hartshorn KL (2013). The human cathelicidin LL-37 inhibits influenza A viruses through a mechanism distinct from that of surfactant protein D or defensins. J Gen Virol.

[26] Lai Y, Adhikarakunnathu S, Bhardwaj K, Ranjith-Kumar CT, Wen Y, Jordan JL (2011). LL37 and cationic peptides enhance TLR3 signaling by viral double-stranded RNAs. PLoS One.

[27] Singh D, Qi R, Jordan JL, San Mateo L, Kao CC (2013). The human antimicrobial peptide LL-37, but not the mouse ortholog, mCRAMP, can stimulate signaling by poly(I:C) through a FPRL1-dependent pathway. J Biol Chem.

[28] Groskreutz DJ, Monick MM, Powers LS, Yarovinsky TO, Look DC, Hunninghake GW (2006). Respiratory syncytial virus induces TLR3 protein and protein kinase R, leading to increased double-stranded RNA responsiveness in airway epithelial cells. J Immunol.

[29] Rudd BD, Burstein E, Duckett CS, Li X, Lukacs NW (2005). Differential role for TLR3 in respiratory syncytial virus-induced chemokine expression. J Virol.

[30] Hotard AL, Lee S, Currier MG, Crowe JE, Sakamoto K, Newcomb DC (2015). Identification of residues in the human respiratory syncytial virus fusion protein that modulate fusion activity and pathogenesis. J Virol.

[31] Johnson PR, Spriggs MK, Olmsted RA, Collins PL (1987). The G glycoprotein of human respiratory syncytial viruses of subgroups A and B: extensive sequence divergence between antigenically related proteins. Proc Natl Acad Sci USA.

[32] Stokes KL, Chi MH, Sakamoto K, Newcomb DC, Currier MG, Huckabee MM (2011). Differential pathogenesis of respiratory syncytial virus clinical isolates in BALB/c mice. J Virol.

[33] Tebbey PW, Hagen M, Hancock GE (1998). Atypical pulmonary eosinophilia is mediated by a specific amino acid sequence of the attachment (G) protein of respiratory syncytial virus. J Exp Med.

[34] Ioannidis I, Ye F, McNally B, Willette M, Flano E (2013). TLR expression and induction of type I and type III interferons in primary airway epithelial cells. J Virol.

